# Intramuscular Injection of Adenoassociated Virus Encoding Human Neurotrophic Factor 3 and Exercise Intervention Contribute to Reduce Spasms after Spinal Cord Injury

**DOI:** 10.1155/2019/3017678

**Published:** 2019-03-11

**Authors:** Yu-Xin Chang, Yan Zhao, Su Pan, Zhi-Ping Qi, Wei-Jian Kong, Yi-Ran Pan, Hong-Ru Li, Xiao-Yu Yang

**Affiliations:** ^1^Department of Orthopedics, Second Hospital of Jilin University, Changchun, China; ^2^Physical Examination Center, Second Hospital of Jilin University, Changchun, China; ^3^Department of Ophthalmology, Second Hospital of Jilin University, Changchun, China

## Abstract

Limb spasms are phenomena of hyperreflexia that occur after spinal cord injury. Currently, the clinical treatment is less than ideal. Our goal is to develop a combination therapy based on individualized medicine to reduce spasticity after spinal cord injury. In this study, rats received a severe contusive injury at the T9 segment of the spinal cord, followed by gene therapy with adenoassociated virus encoding human neurotrophic factor 3 (AAV-NT3) and a 2-week exercise program starting at 4 weeks after injury. We quantified the frequency of spasms during a swimming test at 4 and 6 weeks after injury and confirmed the results of the swimming test by measuring the H-reflex of the plantar muscle. We obtained weekly hind limb exercise scores to assess the effect of the interventions in hind limb motor function improvement. Then, we used immunofluorescence to observe the immunoreactivity of spinal motor neurons, synaptophysin, cholinergic interneurons, and GABAergic interneurons. We also measured the expression of KCC2 in the spinal cord by western blot. We found that AAV-NT3 gene therapy, exercise, and combination therapy all attenuated the frequency of spasms in the swimming test conducted at 6 weeks after spinal cord injury and increased rate-dependent depression of H-reflex. Combination therapy was significantly superior to AAV-NT3 alone in protecting motor neurons. Recovery of KCC2 expression was significantly greater in rats treated with combination therapy than in the exercise group. Combination therapy was also significantly superior to individual therapies in remodeling spinal cord neurons. Our study shows that the combination of AAV-NT3 gene therapy and exercise can alleviate muscle spasm after spinal cord injury by altering the excitability of spinal interneurons and motor neurons. However, combination therapy did not show a significant additive effect, which needs to be improved by adjusting the combined strategy.

## 1. Introduction

Limb spasticity is one of the most common complications after spinal cord injury. It has been reported that 12%–37% of patients with acute spinal cord injury have spasms, and the incidence of limb spasms in patients with chronic spinal cord injury is 65%–78% [1]. The symptoms of spasticity include muscle hypertonia, hyperstimulation of the body, clonus, and muscle spasms accompanied with severe pain [2]. These symptoms are usually caused by peripheral stimulation, such as muscle stretching or tactile stimulation, resulting in an increased myoelectrical response in the skin. This myoelectrical response in the skin in turn results in hyperreflexia of the spinal cord [3]. At present, there is still no effective method to treat the cause of spasms, only to reduce spasm symptoms. Treatments such as tizanidine, baclofen, and botulinum toxin can only temporarily relieve symptoms and have different degrees of side effects [4, 5]. Therefore, the development of a new antispasmodic therapy and a rehabilitation model is essential for the functional recovery and quality of life of patients with spinal cord injury [3]. A single treatment may improve a particular function, but a combination of treatments based on personalized medicine is expected to achieve better results [6–9]. We chose to focus on treatments that improve motor neuron and interneuron survival and function. These include gene therapies that increase the expression of growth factors, as well as exercise, which also improves motor and neuronal function. We therefore investigated the effects of a combination of neurotrophin-3 (NT-3) treatment with exercise, to determine if it would be more efficacious than either therapy alone.

We specifically selected NT-3 for this study given its role in the regeneration of neurons, a role also played by exercise [10]. NT-3 reduces motor neuron excitability, maintains sensory and motor neuron survival, promotes nerve cell differentiation, induces axon growth, and participates in nerve repair after injury [11–17]. The input of afferent signals from peripheral muscles is essential for the recovery of motor function and the reconstruction of neural circuits in spinal cord injury segments [18–22], possibly because peripheral muscle spindles synthesize NT-3 [23]. Kathe et al. showed that overexpression of NT-3 in muscles rebalances excitatory and inhibitory inputs [13]. The same researchers showed that peripheral treatment with recombinant NT-3 improves motor function and neurophysiological outcomes in a rat model of upper limb spasticity following cortical spinal cord injury [13]. However, another study revealed that NT-3 reduces spasticity and normalizes spinal cord reflexes only in spasms caused by stroke and cortical spinal cord injury; its effect in rats with spinal contusions has not been demonstrated [24].

In addition to gene therapy, more basic interventions are also successful in nerve and motor therapy. Exercise is considered the simplest, safest, and most effective treatment method in the clinic [25, 26], which can promote the recovery of sensory and motor function and improve the quality of life of patients [10, 27, 28]. Frigon and Rossignol suggested that exercise improves motor function by balancing the sensory input and motor output functions [29]. Côté et al. found that intensive training normalized proprioceptive reflexes in the spinal cord [20]. A recent study has shown that treadmill training can reduce muscle spasms after spinal cord injury in rats [30]. Our previous study found that functional training can effectively promote axon germination and extension after cortical spinal cord injury as well as the formation of a new circuit by reducing neuronal apoptosis [31].

We aimed to test the effects of a combination of gene therapy and exercise intervention in the reduction of spasms after spinal cord injury. In this study, spinal contusion in rats was followed by a sequential program of peripherally delivered NT-3 and exercise. We then characterized the recovery of motor and neural function with or without these treatments and explored the pathological and pathophysiological mechanisms involved. Studies have shown that the mechanism of spasms includes increased activity and connectivity between proprioceptive afferent neurons and motor neurons, decreased presynaptic inhibition of Ia afferent neurons by spinal cord interneurons, and intrinsic changes in motor neurons such as transport protein concentration on the membrane. Here, we established a rat model of severe spinal cord contusion, which should be sufficient to cause changes in the connectivity between proprioceptive afferents and motor neurons, leading to significant paralysis. Our study confirmed that combination therapy normalizes spinal cord reflexes by balancing excitatory and inhibitory networks in the spinal cord.

## 2. Materials and Methods

### 2.1. Experimental Animals

**Figure 1:**
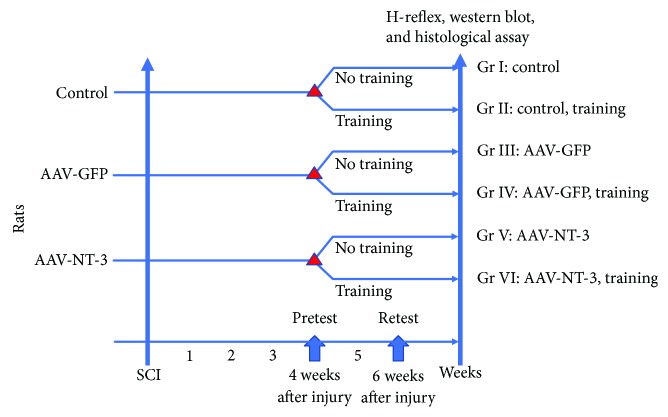
Experimental scheme. Rats were randomized into three groups and received a 250-kilodyne contusive spinal cord injury. Three groups of animals were injected with normal saline, AAV-NT3, or AAV-GFP in the lower limb muscles after spinal cord injury. Three animals in each group were used to detect the expression of NT-3 in the injected lower limb muscle and dorsal root ganglion by western blot at 4 weeks after spinal cord injury. Then, all rats were subjected to a swim test at 4 weeks after spinal cord injury to evaluate the spasticity of the animals. Each group of animals was then divided into two groups: one with exercise and the other without. All rats were retested after 2 weeks for swimming. H-reflex and tissue fixation followed by western blot analysis were performed 1 day later.

Ninety female Wistar rats, each weighing 200–250 g, were obtained from the Experimental Animal Center of Jilin University, China (license number SCXK (Ji) 2008-0005). All rats were housed in standard animal rooms for 1 week prior to surgery to allow acclimatization to housing conditions after being shipped from the facility from which they were obtained. Rats were randomly divided into six groups (Figure 1): control (*n* = 14), simple training (*n* = 14), AAV-GFP (*n* = 14), AAV-GFP + exercise (*n* = 14), AAV-NT-3 (*n* = 14), and AAV-NT3 + exercise (*n* = 14). Control animals received spinal cord contusion surgery without any further intervention. Sham surgery consisted of a procedure in which the lamina was cut without injuring the spinal cord. The research protocol was approved by the Animal Ethics Committee of Jilin University.

### 2.2. Adenoassociated Viral Vectors and Injection of AAV-NT3

The following genes, driven by the human CMV promoter, were inserted into AAV vectors: beta-galactosidase (LacZ), farnesylated green fluorescent protein (f-GFP), and neurotrophin-3 (NT-3). The vector plasmids were transiently transfected into HEK293 cells (ATCC) using calcium phosphate (C0508; Beyotime Biotechnology, China), and the p-AAV/Ad8 and pXX6 helper plasmids generated recombinant AAV serotype 1 carrying the gene of interest. The viruses were purified by heparin affinity chromatography, and the titer was determined by quantitative polymerase chain reaction (qPCR) [24]. AAV-NT3 or AAV-GFP (30 *μ*L; 3.5 × 1011 genome copies) was injected into each rat's bilateral tibialis anterior and soleus muscles at 24 h after spinal cord injury.

### 2.3. Spinal Cord Injury

Rats were initially anesthetized with 5% isoflurane and then continuously exposed to 2% isoflurane during surgery. The hair on the back of each rat was shaved and cleaned with iodine following initial anesthesia. The back skin was incised with a surgical blade, and the subcutaneous soft tissue was bluntly dissected. The paravertebral muscles on both sides of the T8 vertebrae were incised and removed. A dorsal laminectomy of the T8 vertebra was performed using a lamellar bone rongeur to expose the T9 spinal segment. A 250-kilodyne (kdyn) contusion injury was produced using a commercially available spinal cord impactor (IH Impactor, Precision Systems and Instrumentation, VA, USA). The incision site was then cleaned with 0.9% saline, and the researcher ensured that hemostasis was adequate. Paraspinal muscles and surgical incisions were sutured using 4-0 absorbable sutures (Jinhuan, Shanghai, China). Rats were placed on a heating blanket until they recovered from anesthesia and then housed in a single cage. To control the postoperative pain, all animals were treated with penicillin (320,000 units/day for 1 week) and buprenorphine (0.05 mg/kg for 2–3 days). After the surgery, urine was manually squeezed three times a day until the urine function recovered [32].

### 2.4. Swimming Test

**Figure 2:**
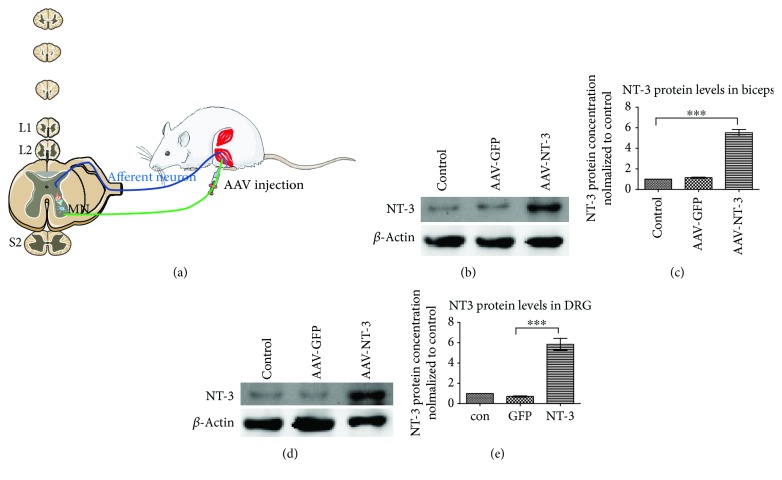
Injection of AAV-NT3 into the gastrocnemius muscle significantly increased NT-3 in muscle and spinal dorsal root ganglia. (a) The schematic diagram shows the experimental setup. Rats received contusive spinal cord injury and injection of AAV-NT3 or AAV-GFP into hind limb muscles. The proprioceptive reflex of the Ia afferent nerve-mediated single synapse is also shown in the diagram. (b) At 4 weeks after spinal cord injury, the expression of NT-3 in the gastrocnemius and tibialis anterior muscles of hind limbs was detected with western blot. (c) Quantitative analysis of the data in (b) showed that the expression of NT-3 in the muscles of the AAV-NT3 group was significantly higher than in the other two groups. (d) At 4 weeks after spinal cord injury, the expression of NT-3 in the spinal cord L4 to S2 DRG was detected by western blot. (e) Quantitative analysis of the data in (d) showed that the expression of NT-3 in the spinal cord DRG of the AAV-NT3 group was significantly higher than in the other two groups. Data are expressed as the mean ± SD. One-way ANOVA was used to compare AAV-NT3 with AAV-GFP or the control group. ^∗∗∗^*p* < 0.05.

According to a protocol by Ryu et al. [33], a swim test was used to observe the spasms occurring after spinal cord injury. The swim test was performed in a rectangular plexiglass chamber (150 cm × 14.5 cm × 40 cm) filled with water to a height of approximately 30 cm. The water temperature was maintained at a constant temperature of 23°C (the most suitable temperature for rat swimming experiments) [2]. Ten swim tests were performed for each rat at both 4 and 6 weeks after spinal cord injury (Figure 2). If more than one spasm or clonus state was observed during swimming, it was recorded as a positive spasm. The percentage of positive spasms in each rat's 10 tests was defined as the frequency of spasm behavior and was recorded. The frequency of the occurrence of spasms was averaged for all rats in each experimental group and was recorded as the frequency of occurrence of spasms in that group.

### 2.5. Behavior Scoring

The Basso, Beattie, and Bresnahan (BBB) scale of 0 to 21 was used to assess the motor function of the hind limbs of rats as described previously [34]. During the 3 min behavioral testing process, the researchers were unaware of the experimental condition of the animals.

### 2.6. Locomotor Training

Rats were trained on a treadmill (7 m/min, 30 min/day; Zhenghua Biotechnology, China) once a day from weeks 4 to 6 after surgery (Figure 1). In order for the injured rat to place the hind paw on the treadmill, we used a safety strap attached to the treadmill to support its weight. Weight support was adjusted to approximately 70% of the animal's weight. When the rat rolled or dragged the hind limbs, the researchers restored the rat to its original position [32].

### 2.7. Hoffmann Reflex (H-Reflex) and Rate-Dependent Depression (RDD) Recordings

The rats were anesthetized with 10% chloral hydrate (3 mL/kg), the distal nerves of the tibia were exposed, and the bipolar cuff was hooked on the level nerves of the ankle. A pair of recording electrodes was then inserted into the plantar muscle of the ipsilateral hind limb [35–37]. We stimulated nerves with stimulating electrodes of 0.2, 0.5, 1, 2, and 5 Hz to measure the RDD of the H-reflex. The average peak value of 15 waveforms was taken as the amplitude of the *M*-wave and *H*-wave under the current stimulus. Rate-dependent changes at each frequency were converted into percentages of the amplitude under 0.2 Hz stimulation.

### 2.8. Perfusion, Fixation, and Immunofluorescence Staining of Spinal Cord Sections

Rats were deeply anesthetized with 10% chloral hydrate solution and fixed by perfusion of the heart with 0.9% saline followed by a 4% poly-A (PFA) solution. The lumbar segment of the spinal cord (L4–L6) was taken and fixed in a 4% PFA solution at 4°C for 24 h, followed by dehydration in a 30% sucrose solution for 3 days. The tissue was cut transversely into 30 *μ*m thick sections on a cryostat. Subsequent antigen retrieval was performed with EDTA for 15 min. After cooling to 50°C, sections were washed three times with 0.01 M phosphate-buffered saline (PBS) and blocked with 10% goat serum for 30 min. Samples were then incubated overnight at 4°C in primary antibody (goat anti-cholinergic acetyltransferase (anti-ChAT), 1 : 100, Abcam, #181023; mouse anti-synaptophysin (SYP), 1 : 100, Abcam, #32127; or rabbit anti-GAD65, 1 : 200, Abcam, #ab11070) mixed with 0.3% Triton X-100. The next day, sections were washed three times with 0.01 M PBS and incubated for 1 h at room temperature with fluorescent secondary antibodies (Alexa Fluor 488 and 568 for each species-matched type, 1 : 200, Beyotime Biotechnology) and DAPI (4′,6-diaminido-2-phenylindole, 1 : 1000, Sigma). After washing, the images were mounted with 50% glycerol and imaged with a confocal microscope (Olympus FV100).

### 2.9. Quantification of Immunofluorescence

The area of immunoreactivity was measured using ImageJ software (v1.51, NIH, USA). A total of 10 sections of the L4 to L6 segments of the spinal cord of each rat were collected for neuronal counting. ChAT-positive cells in the IX layer of the ventral horn in each section having a cell body diameter of more than 40 *μ*m were considered to be motor neurons. The number of motor neurons on both sides of the ventral angle of each slice was measured. The average number of motor neurons in 10 slices was calculated, and the number of motor neurons in each rat was used for statistical analysis. For the calculation of the size of motor neurons, we manually selected the neurons with a diameter greater than 40 *μ*m in the image (0.19 *μ*m per pixel, 640 × 640 pixels) to calculate the size and used Excel for statistical analysis. To quantify SYP, we measured the number of SYP puncta around the cell membrane of 15 motor neurons per rat and averaged them for statistical analysis [38]. ChAT and GAD65 staining were used to detect a positive immune response of interneurons in the VII layer in each section [39]. The number and size of cholinergic interneurons and GABAergic interneurons in each section were calculated, and the average value of each animal was calculated for statistical analysis. To calculate the number and average size of GABAergic interneurons, we converted the image (1.58 pixels per *μ*m, 307 × 307 *μ*m) into black and white binary and adjusted the threshold, then used the watershed function to separate adjacent neurons into a relatively large fluorescent signal. GAD65-positive signals were quantified by setting the analyzed particle submenu to size (*μ*m^2^) 100 infinity at a circularity of 0.00–1.00. The results of all images were statistically analyzed by Excel, and the average value was taken to represent the experimental results of the group. The number and size of ChAT interneurons were calculated in a similar way to those of GABAergic interneurons, except that the calculated image regions (5.2 pixels per *μ*m, 123 × 89 *μ*m) were different.

### 2.10. Western Blot

The expression of NT-3 protein in the muscle and dorsal root ganglia of each group was detected by western blot analysis at 4 weeks after spinal cord injury. The expression of the K^+^/Cl^−^ cotransporter KCC2 protein in the spinal cord tissue of each group was detected by western blot analysis at 6 weeks after spinal cord injury. Briefly, rats were perfused with PBS under anesthesia, and the L5 spinal cord segment (2.5 mm long) was taken. This section was lysed in RIPA buffer (Sigma-Aldrich) containing a 2% protease inhibitor cocktail (Roche, Germany). After centrifugation at 12,000 rpm for 20 min at 4°C, the supernatant was collected and stored at −80°C for western blotting. Protein concentration was determined using the BCA Protein Assay Kit (Thermo Scientific™ Pierce). Equal amounts of protein (20 *μ*g) were separated on a 12% sodium dodecyl sulfate- (SDS-) polyacrylamide gel using electrophoresis and wet transferred to a PVDF membrane (Millipore, MA, USA). The membrane was rinsed in 1x TBST, blocked in 5% bovine serum albumin (BSA) blocking buffer for 1 h at room temperature, and incubated overnight at 4°C with the following primary antibodies: rabbit anti-NT-3 (10 *μ*g/mL, Abcam, ab53685) or rabbit anti-KCC2 (1 : 500, Abcam, ab134300). After washing with TBST, membranes were incubated with horseradish peroxidase- (HRP-) conjugated rabbit IgG (1 : 5000, Invitrogen Life Technologies, Carlsbad, CA, USA) for 1 h at room temperature. To visualize the immunoreactive protein, an enhanced ECL kit (CWBIO, Beijing, China) was used according to the manufacturer's instructions. Quantitative densitometry of captured images was analyzed using ImageJ.

### 2.11. Statistical Analysis

We performed statistical analysis using GraphPad Prism5 software. All results were expressed as the mean ± standard error of the mean (SEM), and *p* < 0.05 was considered to be statistically significant. The Kruskal–Wallis *H* test was used to analyze the BBB score data. Data between groups were compared by unpaired *t*-test or one-way analysis of variance (ANOVA) with the post hoc Bonferroni test or Tukey honest significance difference (HSD) test.

## 3. Results

### 3.1. After Injection of AAV-NT3, the Level of NT-3 in Muscle and Spinal Dorsal Root Ganglia Increased Significantly (Figure 2)

Figure 2(a) shows a schematic diagram of the adenovirus injection procedure. We determined the level of NT-3 in the muscle and the corresponding spinal segment dorsal root ganglia (DRG) by western blot at 4 weeks after spinal cord injury (Figures 2(b) and 2(d)). In the group of rats in which AAV-NT3 encoding human NT-3 was injected into the gastrocnemius muscle, the level of human NT-3 was significantly increased in the ipsilateral muscle or ipsilateral DRG (*p* < 0.001; one-way ANOVA with the post hoc Bonferroni test; Figures 2(c) and 2(e)).

### 3.2. The Effect of AAV-NT3, Exercise, or a Combination of Both in Spasticity Behavior during Swimming (Figure 3)

**Figure 3:**
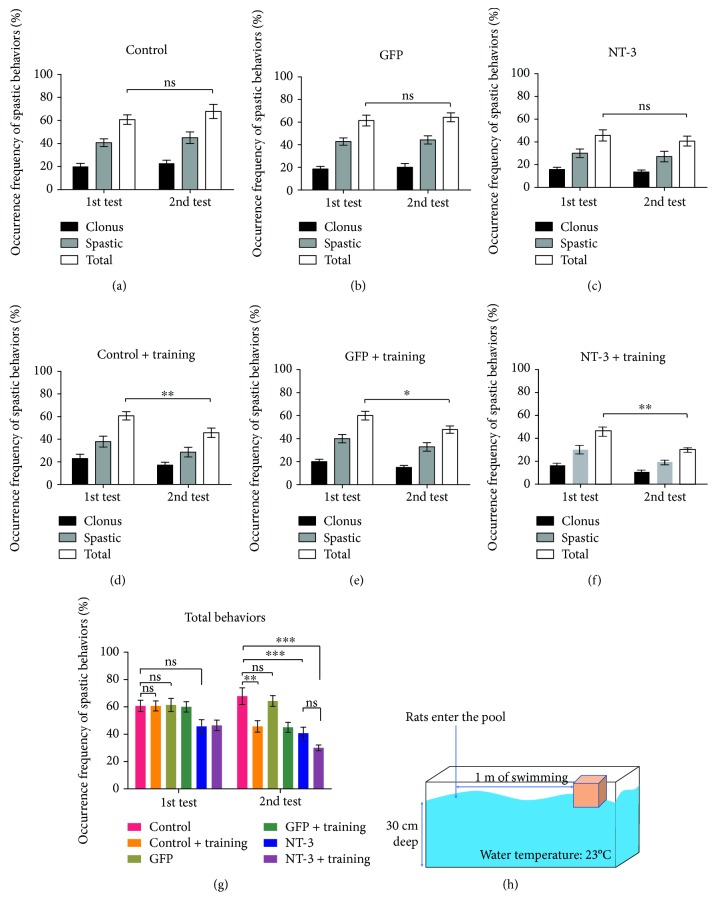
AAV-NT3 and exercise alleviate the spastic behavior in the swimming test. (a–f) The bar graph shows the average frequency of spastic behavior in swimming tests after spinal cord injury in each group, including clone and spastic phases. The first and second tests were performed at weeks 4 and 6 after spinal cord injury, respectively. (g) The bar graph shows the average frequency of total spastic behavior for each experimental group in two tests. (h) Schematic diagram of the swimming test. Data are expressed as the mean ± S.E.M. Unpaired *t*-test or one-way ANOVA was used, and each experimental group was compared with the control group. ^∗^*p* < 0.05, ^∗∗^*p* < 0.01, and ^∗∗∗^*p* < 0.001. ns: nonsignificant.

Figure 3(h) shows a schematic of the swim test, which we used to investigate the effect of individual and combination therapies in spasticity behavior after spinal cord injury in rats. We performed the first swim test at 4 weeks after spinal cord injury and a second swim test at 6 weeks after spinal cord injury (after 2 weeks of exercise). The total spasm frequency during the swim test after exercise was significantly lower than that before exercise (*p* < 0.05, unpaired *t*-test; Figures 3(d)–3(f)). However, the total frequency did not change in the group without exercise intervention (*p* > 0.05; Figures 3(a)–3(c)).

At 6 weeks after spinal cord injury, the total spasm frequency was significantly lower in the simple exercise group compared with the control group (*p* < 0.01; Figure 3(g)). The total spasm frequency was significantly lower in the AAV-NT3 group and the combination group compared with the control group (*p* < 0.001; Figure 3(g)). However, there were no differences between the AAV-NT3 group and the combination group (*p* > 0.05; Figure 3(g)).

### 3.3. AAV-NT3 and Combination Therapies Significantly Improved Motor Function of Hind Limbs (Figure 4)

**Figure 4:**
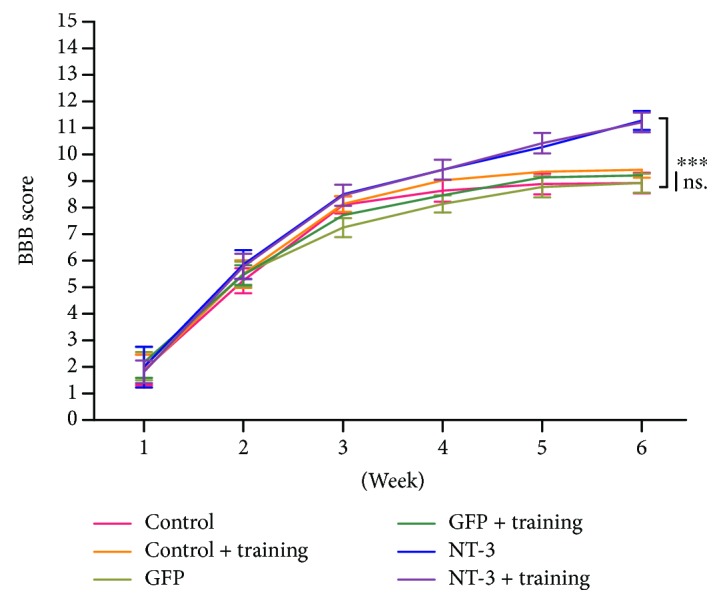
AAV-NT3 and combination therapy significantly improved hind limb motor function. The line graph shows the BBB score for each group within 6 weeks after spinal cord injury. Data are expressed as the mean ± S.E.M. One-way ANOVA was used, and each experimental group was compared with the control group. ns: nonsignificant.

To investigate the effect of individual and combination therapies in improving motor function of the hind limbs, we used the BBB score. The AAV-NT3 group and the combination group significantly improved the motor function of the hind limbs (Kruskal–Wallis test, *p* < 0.05; Figure 4), but there was no statistical difference between the exercise group and the control group (Kruskal–Wallis test, *p* > 0.05; Figure 4).

### 3.4. The RDD of H-Reflex Increased with AAV-NT3 and Exercise, Either Alone or in Combination (Figure 5)

**Figure 5:**
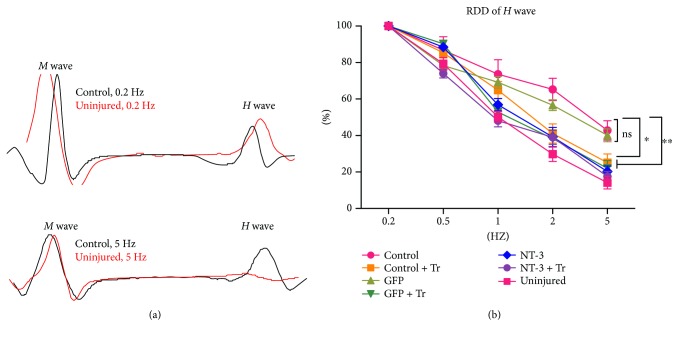
The RDD of H-reflex increased with AAV-NT3 and exercise, either alone or in combination. (a) Sham surgery-treated rats (red line) showed a significant decrease in *H*-wave as the stimulation frequency increased from 0.2 Hz (upper) to 5 Hz (bottom), and this decrease was suppressed in the control group. The *M*-wave did not change in either group. (b) The graph shows the RDD of the *H*-wave of each group of animals. Data are expressed as the mean ± S.E.M. One-way ANOVA was used, and each experimental group was compared with the control group. ^∗^*p* < 0.05, ^∗∗^*p* < 0.01. ns: nonsignificant.

The RDD of the H-reflex is a tool used to assess the degree of spasticity [40–42]. As shown in Figure 5(a), the amplitude of the *H*-wave gradually decreased with increasing stimulation frequency (0.2–5 Hz) in the sham surgery-treated group, while the RDD was significantly reduced in the control group. The *M*-wave remained unchanged as the stimulation frequency changed (Figure 5(a)). When the stimulation frequency was 5 Hz, the RDD was significantly increased in the AAV-NT3 group, the exercise group, and the combination group compared with the control group (one-way ANOVA, post hoc Tukey HSD test, *p* < 0.05; Figure 5(b)). The electrophysiological results were in good agreement with the swim test results, which confirmed the change of the spasticity state after spinal cord injury.

### 3.5. Changes in Motor Neuron Number, Size, and SYP Immunoreactivity after Treatment (Figure 6)

**Figure 6:**
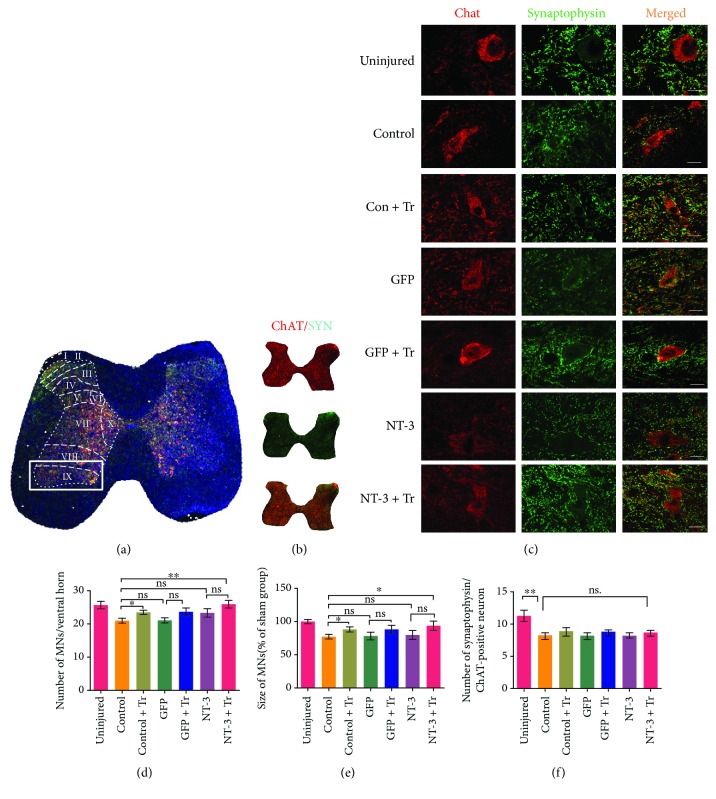
The number of ventral horn motor neurons increased, and the average size increased in the combined treatment group, but there was no significant difference in SYP. (a) Schematic diagram of the spinal cord layer, with the white rectangular box indicating the position of the anterior horn Rexed's lamina IX. (b) Immunofluorescence staining of cholinergic motor neurons ChAT (red) and SYP (green) at low magnification. (c) Expression of ChAT (red) and SYP (green) in ventral horn motor neurons in each group. Scale bar: 20 *μ*m. (d) Bar graph showing changes in the number of motor neurons. (e) Bar graph showing changes in the size of motor neurons. (f) Bar graph showing the average number of SYP puncta in each spinal motor neuron. Data are expressed as the mean ± S.E.M. One-way ANOVA was used, and each experimental group was compared with the control group. ^∗^*p* < 0.05, ^∗∗^*p* < 0.01. ns: nonsignificant.

The motor neurons located in the IX layer of the lumbar spinal cord segment play a role in controlling the muscle activity of the hind limbs. To explore the specific mechanisms by which interventions reduce spasm behavior, we stained motor neurons with anti-acetylcholine antibodies. Univariate ANOVA showed that the exercise group and the combination group increased the number of motor neurons after spinal cord injury (*p* < 0.05; Figure 6(d)) and that the size of these motor neurons increased compared with those in the control group (*p* < 0.05; Figure 6(e)). There were no changes in motor neuron number or size in the AAV-NT3 group. The SYP content of motor neurons in all groups was significantly lower than that in the sham surgery-treated group (*p* < 0.01; Figure 6(f)), but there was no significant difference between the control group and any of the treatment groups (*p* > 0.05; Figure 6(f)).

### 3.6. Combination Therapy, but Not Individual Treatments, Increased the Size of Acetylcholine Interneurons in the Spinal Cord (Figure 7)

**Figure 7:**
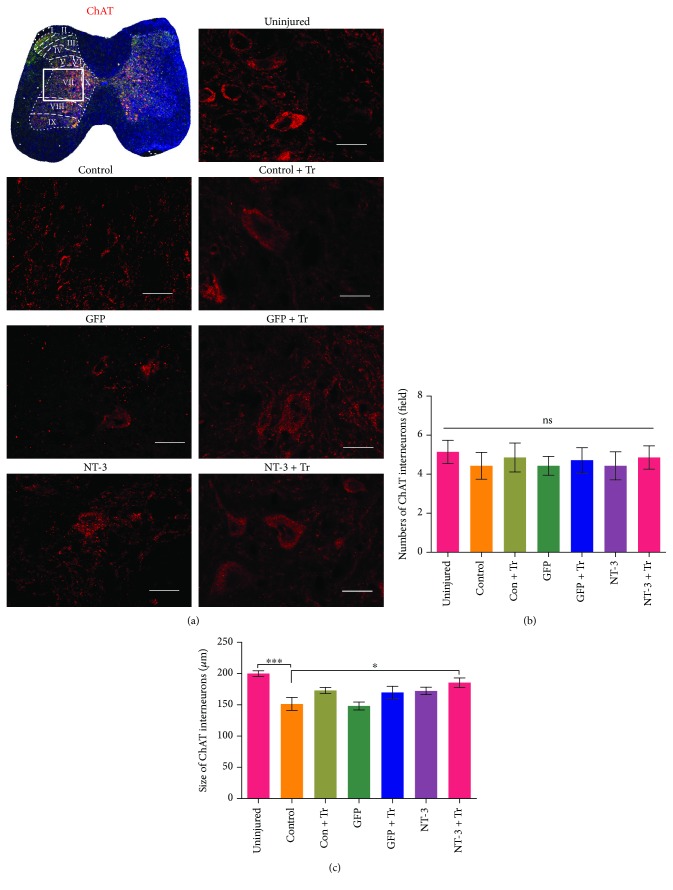
Compared with the sham surgery-treated group, the size of cholinergic interneurons in the central spinal cord gray matter (Rexed's lamina VII) was smaller but the number did not change in each experimental group. (a) Schematic diagram of the spinal layer, with the white rectangular box indicating the position of the VII layer. Representative immunofluorescence staining of cholinergic interneuron ChAT (red) in each group. Scale bar: 20 *μ*m. (b) Bar graph showing changes in the number of cholinergic interneurons in each group. Number of cells counted per field (123 *μ*m × 89 *μ*m). (c) Bar graph showing changes in the size of cholinergic interneurons in each group. Data are expressed as the mean ± S.E.M. One-way ANOVA was used, and each experimental group was compared with the control group. ^∗^*p* < 0.05, ^∗∗^*p* < 0.01. ns: nonsignificant.

We then investigated whether interventions to reduce spasm behavior had an effect on the number or size of cholinergic interneurons. Statistical analysis showed that all interventions did not significantly alter the number of cholinergic interneurons after spinal cord injury compared with the control group (*p* > 0.05; Figure 7(b)). There was no statistical difference in the size of cholinergic interneurons between the control group and any of the individual treatment groups (*p* > 0.05; Figure 7(c)), but that of the combination group was significantly increased (*p* < 0.05; Figure 7(c)).

### 3.7. Combination Therapy Increased the Number of GABAergic Interneurons in the Spinal Cord (Figure 8)

**Figure 8:**
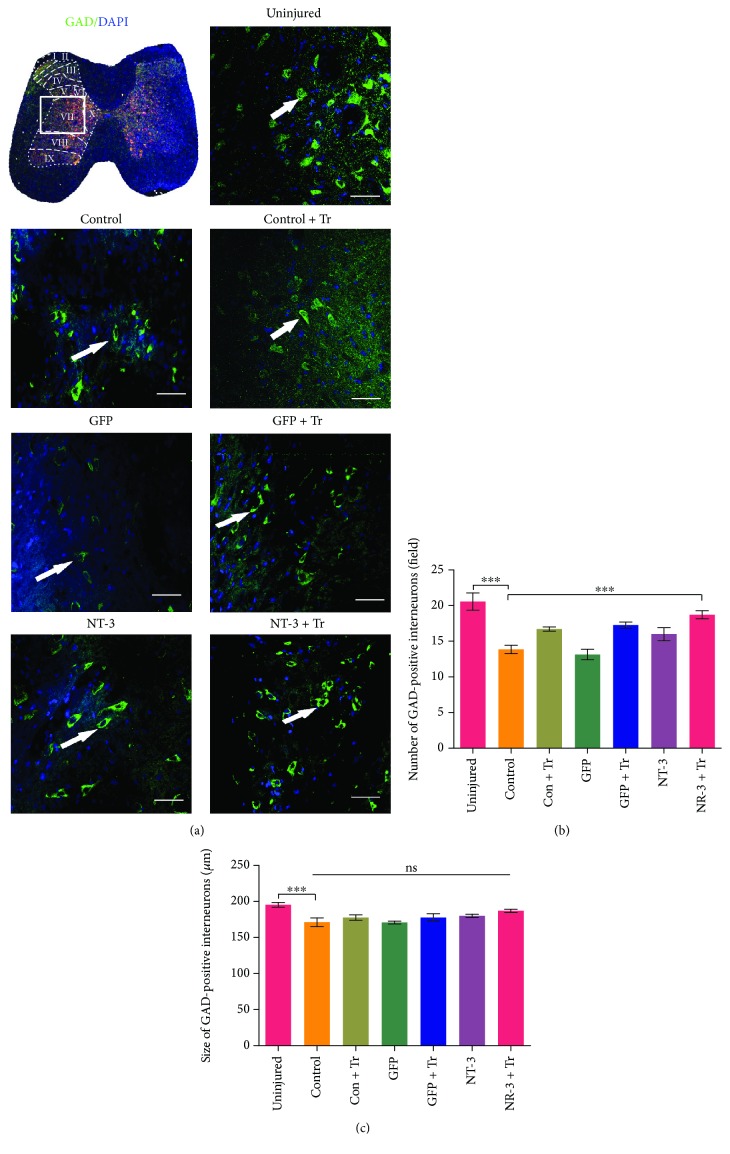
Compared with the sham surgery-treated group, the number of GABAergic interneurons in the central spinal cord gray matter (Rexed's lamina VII) was significantly increased but the size did not change. (a) Schematic diagram of the spinal layer, with the white rectangular box indicating the position of the VII layer. Representative immunofluorescence staining of GAD65-positive GABAergic interneurons (green) in each group. Scale bar: 50 *μ*m. (b) Bar graph showing changes in the number of GABAergic interneurons in each group. Number of cells counted per field (307 *μ*m × 307 *μ*m). (c) Bar graph showing changes in the size of GABA interneurons in each group. Data are expressed as the mean ± S.E.M. One-way ANOVA was used, and each experimental group was compared with the control group. ^∗∗∗^*p* < 0.001. ns: nonsignificant.

Inhibitory GABAergic interneurons are the most important inhibitory interneurons in the spinal cord. To investigate whether inhibitory interneurons changed with interventions to reduce spasm behavior, we used GAD65 antibodies to label inhibitory interneurons in the spinal cord. Statistical analysis showed that combination therapy increased the number of GABAergic interneurons in the spinal cord (*p* < 0.001; Figure 8(b)), while AAV-NT3, exercise, or their combination did not significantly change GABAergic interneuron size (*p* > 0.05; Figure 8(c)).

### 3.8. Combination Therapy Increased the Expression of KCC2 in the Spinal Cord (Figure 9)

**Figure 9:**
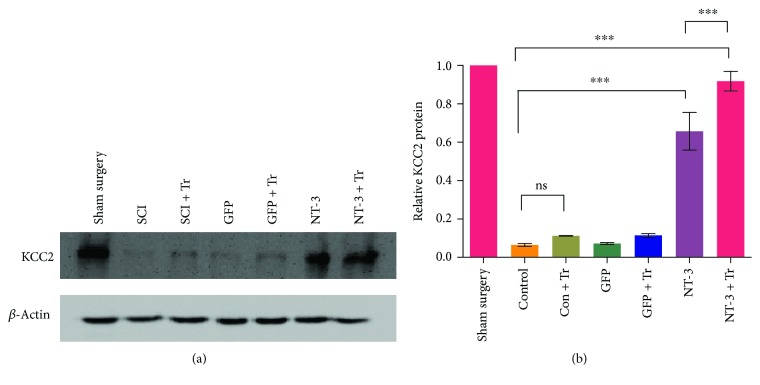
AAV-NT3, exercise, or combination therapy increased the expression of KCC2 in the spinal cord after spinal cord injury. (a) After 6 weeks of spinal cord injury, the expression of KCC2 in the spinal cord L4 to S2 was detected by western blot. (b) Quantitative analysis of the data in (a). Data are expressed as the mean ± S.E.M. One-way ANOVA was used, and each experimental group was compared with the control group. ^∗∗∗^*p* < 0.001. ns: nonsignificant.

KCC2 on the cell membrane lowers the excitability of motor neurons by exporting chloride and potassium ions. KCC2 is reduced in the motor cell membrane after spinal cord injury, which leads to excessive excitation of motor neurons [43, 44]. Statistical analysis showed that AAV-NT3, exercise, and combination therapy significantly increased the content of KCC2 (*p* < 0.001; Figure 9(b)). At the same time, the combination group showed a significant increase in KCC2 levels compared to the exercise group (*p* < 0.001; Figure 9(b)). It is speculated that this may be an important mechanism by which interventions can alleviate paralysis.

## 4. Discussion

This study examined the effect of the sequential therapy of AAV-NT3-based gene therapy and exercise on muscle spasms after spinal cord injury. Previous studies have shown that swim tests have a good correlation with H-reflex RDD and can reliably test the extent of muscle spasms after spinal cord injury [32, 33]. We therefore used a swim test to quantify the frequency of muscle spasms after spinal cord injury and confirmed the results of the swim test by H-reflex RDD. The results showed that AAV-NT3 gene therapy, exercise, and combination therapy can all alleviate muscle spasms after injury. The combination therapy had a trend of a beneficial effect compared to gene therapy or exercise alone, but the difference was not significant. We suspect that this may be related to the time point and duration of each individual therapy. In follow-up studies, we will explore the best way to treat muscle spasms after spinal cord injury. Greater NT-3 expression levels and earlier exercise intervention may have a more effective effect.

In order to explore the specific mechanism by which therapeutic interventions alleviate hyperreflexia in rats, we used immunofluorescence colocalization to study the changes in the number and morphology of motor neurons and the surrounding SYP in the lumbar spinal cord segment. We found that exercise and combination therapy, but not AAV-NT3 gene therapy, can restore the number and size of motor neurons to normal levels. These results indicate that although AAV-NT3 gene therapy and exercise can alleviate spasticity, the mechanism of action of the two is not identical.

Next, we explored a deeper mechanism for the above changes, particularly whether this therapeutic effect affects interneurons. Studies have shown that, after spinal cord injury, the rapid and substantial reduction of cholinergic input to motor neurons and the sharp decrease in presynaptic GABAergic signals on primary afferent fibers of Ia may be the important mechanisms leading to muscle spasms [39, 45–47]. We performed immunofluorescence staining of ChAT-positive cholinergic interneurons and GAD-positive GABAergic interneurons in the VII layer of the spinal cord. We found that the combination therapy had no effect on the number of ChAT interneurons but could significantly change their size to return to normal levels. At the same time, combination therapy significantly increased the number of GAD-positive interneurons. ChAT-positive cholinergic interneurons act as excitatory interneurons that increase the transmission of excitatory signals to motor neurons in lamina IX [39]. At the same time, GAD-positive GABAergic interneurons, the largest population of inhibitory interneurons in the spinal cord, show a significant reduction in the number of layers in the VII layer after spinal cord injury [45, 48–51]. The ratio of excitatory to inhibitory interneurons in the gray matter affects the central nervous circuit in the spinal cord [52–54]. At the same time, it can change the projection of the plane IX layer of the spinal cord, which affects the inhibition of Ia afferent fibers [55–57]. We speculate that this is a key mechanism for the combination therapy to alleviate hyperreflexia in rats. The changes in the interneurons of the VII layer found in our study are in good agreement with the changes in the motor neurons and Ia afferent fibers in the IX layer found in the previous study [13].

Changes in the electrophysiological properties of motor neurons are also one of the important mechanisms leading to paralysis after spinal cord injury [58]. Previous studies have demonstrated that exercise can reduce the excitability of spinal motor neurons after injury by increasing the expression of the KCC2 transporter on the cell membrane of spinal motor neurons [59–61]. Our results indicate that gene therapy with AAV-NT3 can significantly increase the expression of KCC2 and the increase in KCC2 in the combination group was significantly higher than in the exercise group. Our results suggest that combination therapy can affect the spasticity state after injury through different mechanisms.

Although each of the individual therapies and the combination therapy alleviated spasm behavior, significantly improved hind limb motor function was observed only in the AAV-NT3 group and the combination group. This may be related to severe spinal cord contusion surgery that prevented most rats from walking upright after 4 weeks of spinal cord injury, by which time muscle weakness had been established and rats were unable to fully benefit from exercise.

In conclusion, our study suggests that combination therapy can alleviate muscle spasm after spinal cord injury. This effect on hyperreflexia is due to combination therapy that can rebalance excitatory and inhibitory networks in the spinal cord, contributing to the normalization of monosynaptic and multiple synaptic reflexes.

## 5. Conclusion

Our study shows that AAV-NT3 gene therapy and exercise can alleviate muscle spasms after spinal cord injury by changing the excitability of spinal neurons and motor neurons. Although combination therapy did not show significant additive effects, combination therapy was superior to treatment alone in different aspects. The best way to treat muscle spasms after spinal cord injury depends on further exploration.

## Data Availability

The data used to support the findings of this study are available from the corresponding author upon request.
